# A quick and effective trait-based protocol for selecting appropriate native plant species for the reforestation of degraded tropical mines

**DOI:** 10.3389/fpls.2024.1456740

**Published:** 2024-08-14

**Authors:** Changbin Xu, Hui Zhang, Huai Yang, Cui Chen, Chen Wang

**Affiliations:** ^1^ College of International Tourism and Public Administration, Hainan University, Haikou, China; ^2^ Hainan Institute of National Park, Haikou, China; ^3^ Institute of Tropical Bamboo, Rattan & Flower, Sanya Research Base, International Center for Bamboo and Rattan, Sanya, China; ^4^ School of Geography and Tourism, Huanggang Normal University, Huanggang, Hubei, China; ^5^ Department of Agricultural and Environmental Sciences, Tennessee State University, Nashville, TN, United States

**Keywords:** deforestation, human disturbances, trait-based plant species selection protocol, tropical forest ecosystem, reforestation, Hainan Island

## Abstract

**Introduction:**

A critical issue in tropical forests is that anthropogenic deforestation (i.e., mining) degrades the integrity of its ecosystem. Reforestation with appropriate native plant species helps to alleviate these detrimental impacts. A protocol to select appropriate plant species for this purpose currently lacks efficacy and timeliness.

**Methods:**

We provided a trait-based protocol to quickly and effectively select native plant species for mining reforestation. A 0.2-km^2^ area of Baopoling (BPL) at Hainan Island, China, was used as a study site, which has been severely degraded by 20 years of limestone mining for cement production. First, we identified the tree species in nearby undisturbed tropical forests, followed by evaluating the similarities in functional traits of the most dominant one (target species) and 60 local candidate native plant species (candidate species) whose saplings can be purchased from a local market.

**Results and discussion:**

This dataset was used in our trait-based protocol, and only within 1 month, we successfully selected eight plant species which are very similar to target species from the 60 candidate species. We also quantified whether the eight selected plant species were indeed suitable for sustained reforestation by testing their effects on landscape and also their survival rate and recruitment ability after using them to perform reforestation in BPL from 2016 to 2023. Finally, these eight plant species are indeed suitable for reforestation due to their huge influences on a significant shift from originally degraded landscape (comprising only barren rocks) to a forest landscape totally and also their high survival rate (90%–97%) and ability for natural recruitment after 7 years’ reforestation in BPL. Thus, we anticipate that this protocol would be integral to species selection during reforestation of tropical mining areas.

## Introduction

Disproportionate human disturbances (e.g., ore mining and agricultural use) potentially lead to an extensive deforestation of tropical forests (Laurance et al., 2014). This degradation manifests into global biodiversity loss, increases carbon emissions, and water loss (Aleman et al., 2018; Mitchard, 2018; Symes et al., 2018; Taubert et al., 2018). Industrial concessions for logging and plantations (e.g., for fiber, and oil palm) may explain the high ratios of forest loss compared with their gain in the tropics. However, mining is increasingly identified as an important driver of deforestation (Butsic et al., 2015; González-González et al., 2021). Yet, mining is rarely listed as a major cause of forest loss in the global or regional assessments (Abood et al., 2015). As a result, the true extent and impacts of mining may be significantly underestimated (Sonter et al., 2017; Siqueira-Gay and Sánchez, 2021).

There is little doubt, however, that mining is a rapidly growing, highly destructive industrial activity, and a significant contributor to forest loss and land degradation (Ahmed et al., 2021). The rapid removal of vegetation and soils and the overburden of rock substrates that remain after mining make the vegetation recovery difficult (Macdonald et al., 2015). Restoration of mined-out lands to forests is therefore a major goal of land management (Lamb et al., 2005). In practice, reforestation has been recognized as an effective method to restore mined areas (Ahirwal et al., 2017) and derive biodiversity and ecosystem service benefits (Hua et al., 2022).

Reforestation and forest protection are commonly used to alleviate the detrimental impacts of deforestation (Griscom et al., 2017; Taubert et al., 2018). An important challenge in a successful reforestation exercise is the identification of appropriate native tree species (Brown and Amacher, 1999; Fry et al., 2013; Jones, 2013). A trial-and-error method is usually employed to select the best-fitting tree species from candidate native tree species for reforestation. This requires expert knowledge of historical tree species composition, habitat requirements, and trailing (Packard and Mutel, 1997). Moreover, selected tree species may only be applicable for reforestation in specific ecosystems (Laughlin and Laughlin, 2013). Therefore, a method is needed that can quickly and effectively select several appropriate tree species.

The trait-based species selection framework focuses on quickly and effectively choosing native candidate species for ecological restoration (Laughlin, 2014; Wang et al., 2020). This framework assumes that plant functional traits, including morphological, physiological, and phenological traits, reflect a plant’s life history. Plant species that thrive in a given habitat should therefore possess similar functional traits, to similarly respond to abiotic environmental influences (Funk et al., 2008; Laughlin, 2014). Based on this assumption, tree species with functional traits similar to those thriving in the target ecosystem (e.g., dominant species) should be suitable for reforestation. Indeed, Wang et al. (2020) found that candidate species selected by using the criteria of similarity of functional traits could effectively reforest a degraded island ecosystem. Yet, a study that applies this trait-based species selection method to mining reforestation is still lacking. Moreover, a general quantitative protocol for using such trait-based species selection framework to guide mining restoration is also still needed (Wang et al., 2020). As a result, here we expect to use such a trait-based plant species selection framework to develop a general protocol for quickly and effectively choosing appropriate tree species for mining reforestation. This protocol required the following: a measurement of dominant plant species’ functional traits from nearby undisturbed forests; an identification of candidate species; and the use of the trait-based plant species selection software platform provided in Wang et al. (2020) to designate an appropriate reforestation plan. To assess the efficacy of our protocol, we quantified whether selected plant species could successfully reforest a 0.2-km^2^ study site in Baopoling (BPL), Hainan island, China. A 20-year limestone extraction for cement production had rendered BPL as barren and unforgiving, with no vegetation. Since the selected tree species has colonized and survived in this area, our protocol may have wide applicability in identifying a larger pool of candidate species for reforestation of degraded tropical forest ecosystems across the world.

## Materials and methods

### Study sites

**Figure 1:**
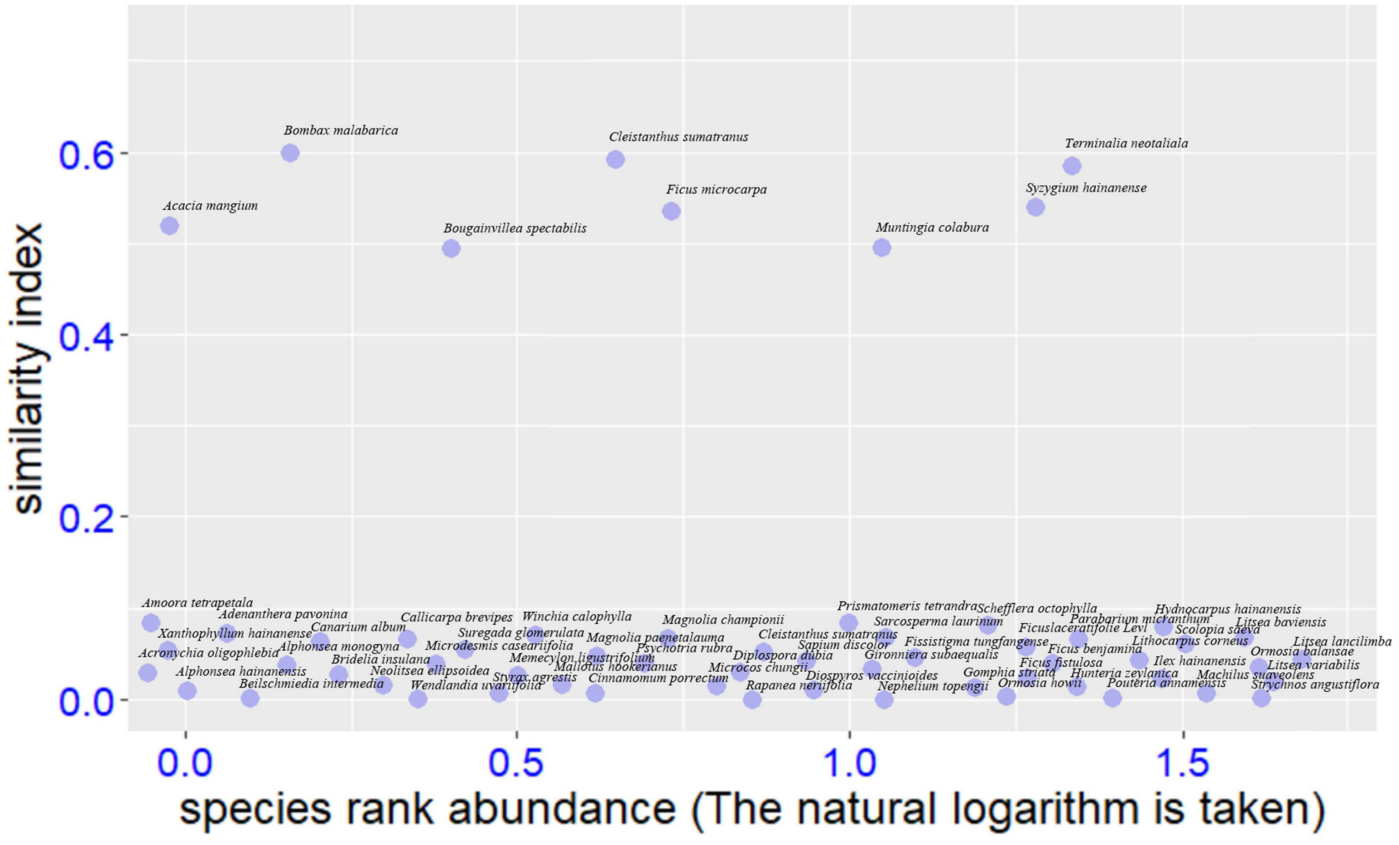
Results of selection of target species, *Bridelia tomentosa*, and 60 candidate native tree species, with similarity index >0.5 indicating appropriate for restoration.

Our study site was located in Baopoling (BPL), on a limestone mountain in Sanya City, Hainan, China (109°51′01"E, 18°31′99"N). It has a tropical monsoon oceanic climate with a mean annual temperature of 28°C. The average annual precipitation on Hainan Island is 1,500 mm, and most (approximately 91%) of the precipitation occurs from June to October (Zhang et al., 2023). Typical vegetation in BPL is composed of tropical monsoon broad-leaf forest. One part (0.2-km^2^ area) of BPL was extensively degraded and lacks vegetation (Zhang et al., 2024). The other parts of BPL remain undisturbed and possess a rich tropical rainforest (**Figure 1**).

### Determining target and candidate tree species

Our protocol requires an identification of target species (nearby native species which are proven appropriate for a degraded ecosystem) as a baseline from which the selection of candidate tree species could be made. If restoration is initiated from bare soil conditions, data measured from dominant plant species in a nearby or similar habitat could be used as a proxy (Ostertag et al., 2015). We therefore selected the most dominant tree species (*Bridelia tomentosa*) in the nearby undisturbed tropical monsoon forests in BPL as our target species. Since our study site lacked any vegetation, restoring this area is comparable with an early forest succession. Fast-growing tree species usually dominate an early succession (Lohbeck et al., 2014). As a result, we selected all the fast-growing candidate native tree species that could be purchased from a local nursery in Sanya City. Finally, we have found 60 candidate tree species (**Table 1**).

**Table 1:** Species list for the 60 fast-growing candidate native tree species that could be purchased from a local nursery in Sanya City.

Species	Family	Genus
*Acacia mangium*	Leguminosae	acacia
*Acronychia oligophlebia*	Rutaceae	Acronychia
*Adenanthera pavonina*	Leguminosae	Adenanthera
*Alphonsea hainanensis*	Annonaceae	Alphonsea
*Alphonsea monogyna*	Annonaceae	Alphonsea
*Amoora tetrapetala*	Meliaceae	Amoora
*Beilschmiedia intermedia*	Lauraceae	Beilschmiedia
*Bombax malabarica*	Malvaceae	Bombax
*Bougainvillea spectabilis*	Nyctaginaceae	Bougainvillea
*Bridelia insulana*	Phyllanthaceae	Bridelia
*Callicarpa brevipes*	Lamiaceae	Callicarpa
*Canarium album*	Burseraceae	Canarium
*Cinnamomum porrectum*	Lauraceae	Cinnamomum
*Cleistanthus sumatranus*	Phyllanthaceae	Cleistanthus
*Cleistanthus sumatranus*	Phyllanthaceae	Cleistanthus
*Diospyros vaccinioides*	Ebenaceae	Diospyros
*Diplospora dubia*	Rubiaceae	Diplospora
*Ficus benjamina*	Moraceae	Ficus
*Ficus fistulosa*	Moraceae	Ficus
*Ficus microcarpa*	Moraceae	Ficus
*Ficuslaceratifolie Levl*	Moraceae	Ficus
*Fissistigma tungfangense*	Annonaceae	Fissistigma
*Gironniera subaequalis*	Cannabaceae	Gironniera
*Gomphia striata*	Ochnaceae	Campylospermum
*Hunteria zeylanica*	Apocynaceae	Hunteria
*Hydnocarpus hainanensis*	Achariaceae	Hydnocarpus
*Ilex hainanensis*	Aquifoliaceae	llex
*Lithocarpus corneus*	Fagaceae	Lithocarpus
*Litsea baviensis*	Lauraceae	Litsea
*Litsea lancilimba*	Lauraceae	Litsea
*Litsea variabilis*	Lauraceae	Litsea
*Machilus suaveolens*	Lauraceae	Machilus
*Magnolia championii*	Magnoliaceae	Magnolia
*Magnolia paenetalauma*	Magnoliaceae	Magnolia
*Mallotus hookerianus*	Euphorbiaceae	Mallotus
*Memecylon ligustrifolium*	Melastomataceae	Memecylon
*Microcos chungii*	Malvaceae	Microcos
*Microdesmis caseariifolia*	Pandaceae	Microdesmis
*Muntingia colabura*	Muntingiaceae	Muntingia
*Neolitsea ellipsoidea*	Lauraceae	Neolitsea
*Nephelium topengii*	Sapindaceae	Nephelium
*Ormosia balansae*	Fabaceae	Ormosia
*Ormosia howii*	Fabaceae	Ormosia
*Parabarium micranthum*	Apocynaceae	Parabarium
*Pouteria annamensis*	Sapotaceae	Pouteria
*Prismatomeris tetrandra*	Rubiaceae	Prismatomeris
*Psychotria rubra*	Rubiaceae	Psychotria
*Rapanea neriifolia*	Primulaceae	Rapanea
*Sapium discolor*	Euphorbiaceae	Sapium
*Sarcosperma laurinum*	Sapotaceae	Sarcosperma
*Schefflera octophylla*	Araliaceae	Schefflera
*Scolopia saeva*	Salicaceae	Scolopia
*Strychnos angustiflora*	Loganiaceae	Strychnos
*Styrax agrestis*	Styracaceae	Styrax
*Suregada glomerulata*	Euphorbiaceae	Suregada
*Syzygium hainanense*	Myrtaceae	Syzygium
*Terminalia neotaliala*	Combretaceae	Terminalia
*Wendlandia uvariifolia*	Rubiaceae	Wendlandia
*Winchia calophylla*	Apocynaceae	Winchia
*Xanthophyllum hainanense*	Polygalaceae	Xanthophyllum

### Evaluation of functional trait

It has been found that various types of traits need to be evaluated for selecting an appropriate species for reforestation (Wang et al., 2021). BPL’s notable dry season favor drought-tolerant plants. In February of 2016, at the peak of the dry season, we evaluated functional traits that are highly related to growth rate and drought stress tolerance. These traits included the following: upper epidermic thickness (μm); palisade tissue (μm); spongy tissue (μm); lower epidermic thickness (μm); stomatal density (numbers mm^−2^); transpiration rate (mol m^-2^ s^−1^); maximum photosynthesis rate (mol m^-2^ s^−1^); stomatal conductance (mmol m^−2^ s^−1^); leaf water conductance (mmol m^−2^ s^−1^ MPa^−1^); specific leaf area (g/cm^2^); leaf dry matter content (%); and leaf turgor loss point (MPa). Measurements were conducted on the target species (*Bridelia tomentosa*) and 60 candidate plant species. There were 20 fully expanded, healthy, and sun-exposed leaves collected from five mature trees of each target and candidate species, all of which possessed a diameter at breast height (DBH) value comparable with the mean DBH values of respective (target and candidate) species. Traits were measured following the methods described in Hua et al. (2017) and Zhang et al. (2018). The detailed procedures for measuring these functional traits are described in the **Supplementary Materials**. All measured traits for target and candidate plant species were further inputted into our following tree species selection software platform to check the similarity between target and candidate plant species.

### Tree species selection software platform

In a previous work, a trait-based plant species selection software platform was developed, which could identify plant species that possess functional traits that are similar to a target plant species from an available candidate pool (Wang et al., 2020). The software platform uses the maximum entropy model developed by Shipley et al. (2006) to calculate the similarity index, which determines whether candidate species are suitable for reforestation. A high similarity index (i.e., >0.5) indicates greater similarity between a candidate and target plant species and thus the candidate species is fit for restoration (Wang et al., 2020). In contrast, a low similarity index (e.g., 0.1–0.2) indicates that the candidate species is not suitable for reforestation (Wang et al., 2020). For further details on our software platform, please see Wang et al. (2020); the Home page of the platform is shown in **Supplementary Figure 1**.

### Survival rates, mean height, and diameter at breast height

Monoculture plantation of fast-growing species can accelerate forest recovery (Lugo, 1997). We monocultured seedlings for all selected tree species in eight reconstructed soil layers in BPL by the end of 2016 (Zhang et al., 2023). There was at least a 3-m interval between any two planted stems. For detailed descriptions of our plantation, please see Zhang et al. (2024). The initial height and DBH for saplings of all selected tree species ranged 1 m–2 m and 1 cm–2 cm, respectively. The planting density was kept at 50–80 stems per hectare. If these selected tree species are really appropriate, they should not only have a high survival rate (Wang et al., 2020) but also can recruit naturally, without requiring manual management (Holl et al., 2017). In addition, the original landscape should also have a big alteration after using selected tree species to perform reforestation. As a result, we recorded their survival rates from 2016 to 2023 as.


$$\text{survival rate}=\frac{\text{Remaining seedings}}{\text{Original seedings}}\times 100\%$$

We also measured the height and DBH for all individuals for all selected tree species in 2023 to find out whether they can naturally recruit. In addition, we compared the landscape change between before reforestation and after reforestation by comparing the differences in remote sensing image.

## Results

**Figure 2:**
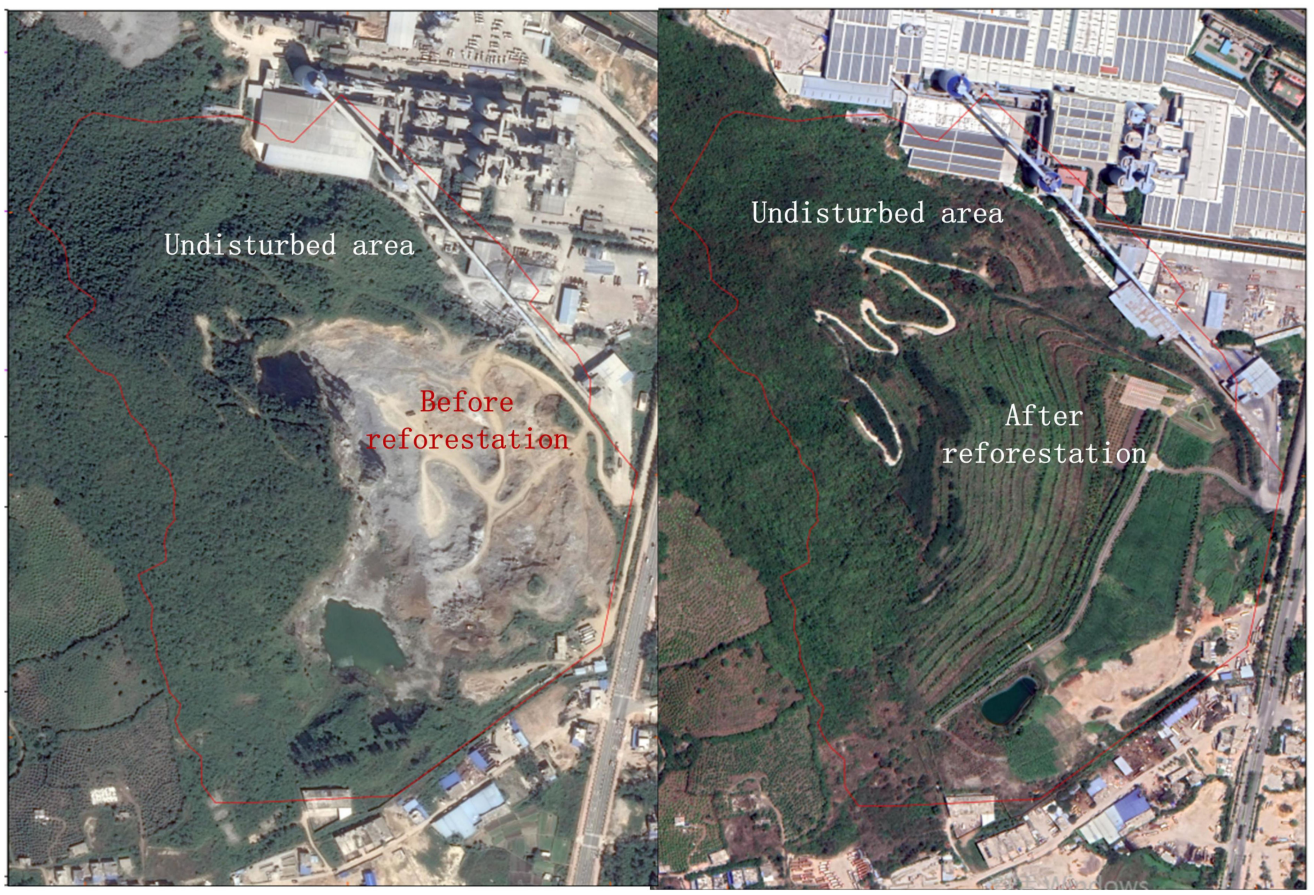
The landscape for extremely degraded and undisturbed tropical monsoon forests in Baopoling (BPL) before reforestation and after reforestation.

Final screening results which are based on using the functional traits to compare the similarity index between target species and candidate species are shown in **Figure 2**. Eight native tree species (*Acacia mangium*, *Bombax malabarica*, *Bougainvillea spectabilis*, *Cleistanthus sumatranus*, *Ficus microcarpa*, *Muntingia calabura*, *Syzygium hainanense*, and *Terminalia neotaliala*) showed very suitable for restoration, as they were very similar to the target species, *Bridelia tomentosa*, which is clearly witnessed by the similarity index for the eight species all being more than 0.5 (**Figure 1**). In contrast, the similarity index for the rest of the 52 candidate native species were from 0.1 to 0.2 (**Figure 1**), indicating they are not appropriate for reforestation.

**Figure 3:**
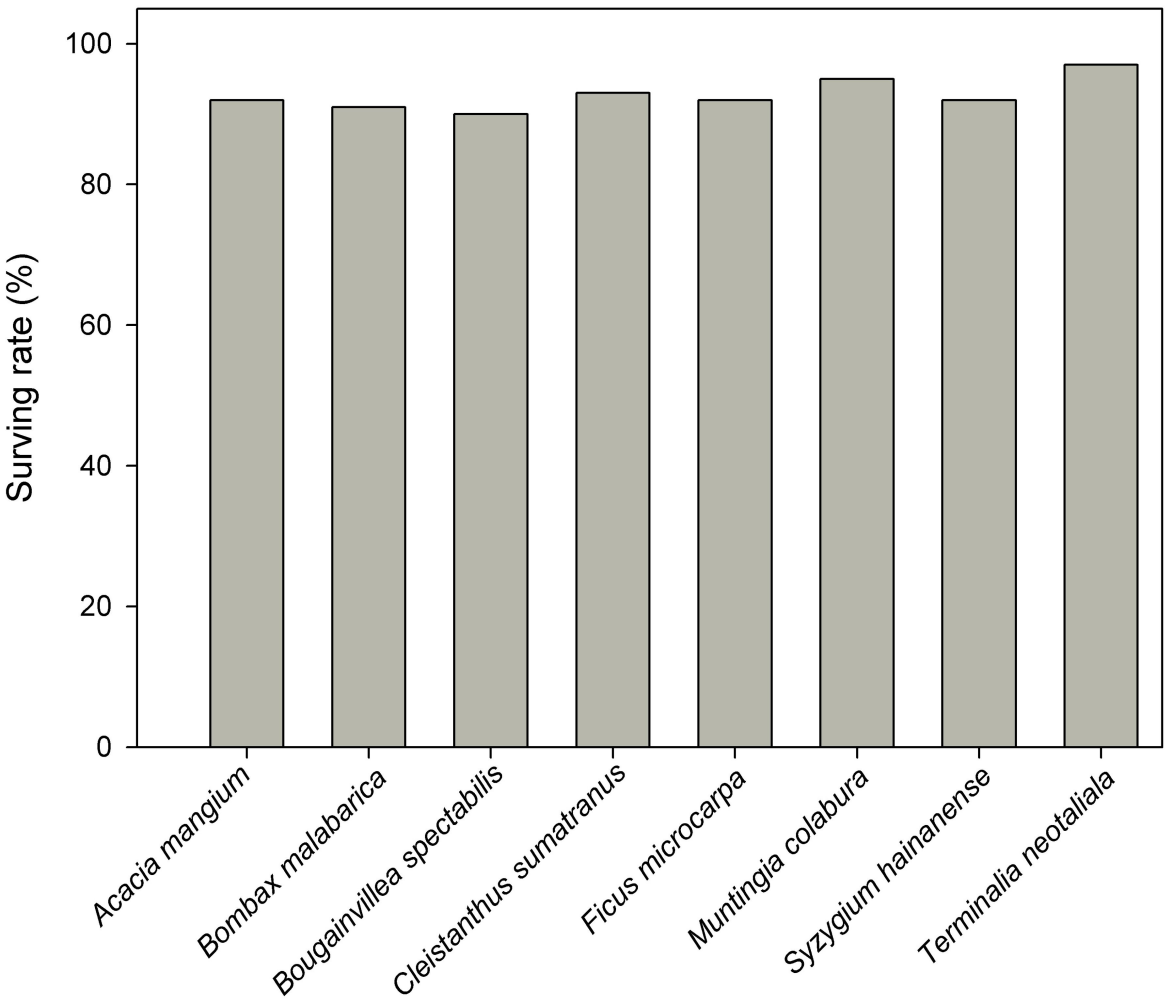
The survival rate of eight selected tree species. Acacia mangium, Bombax malabarica, Bougainvillea spectabilis, Cleistanthus sumatranus, Ficus microcarpa, Muntingia calabura, Syzygium hainanense, and Terminalia neotaliala were used for reforestation in 2016 in BPL. Survival rates were recorded in 2023.

**Figure 4:**
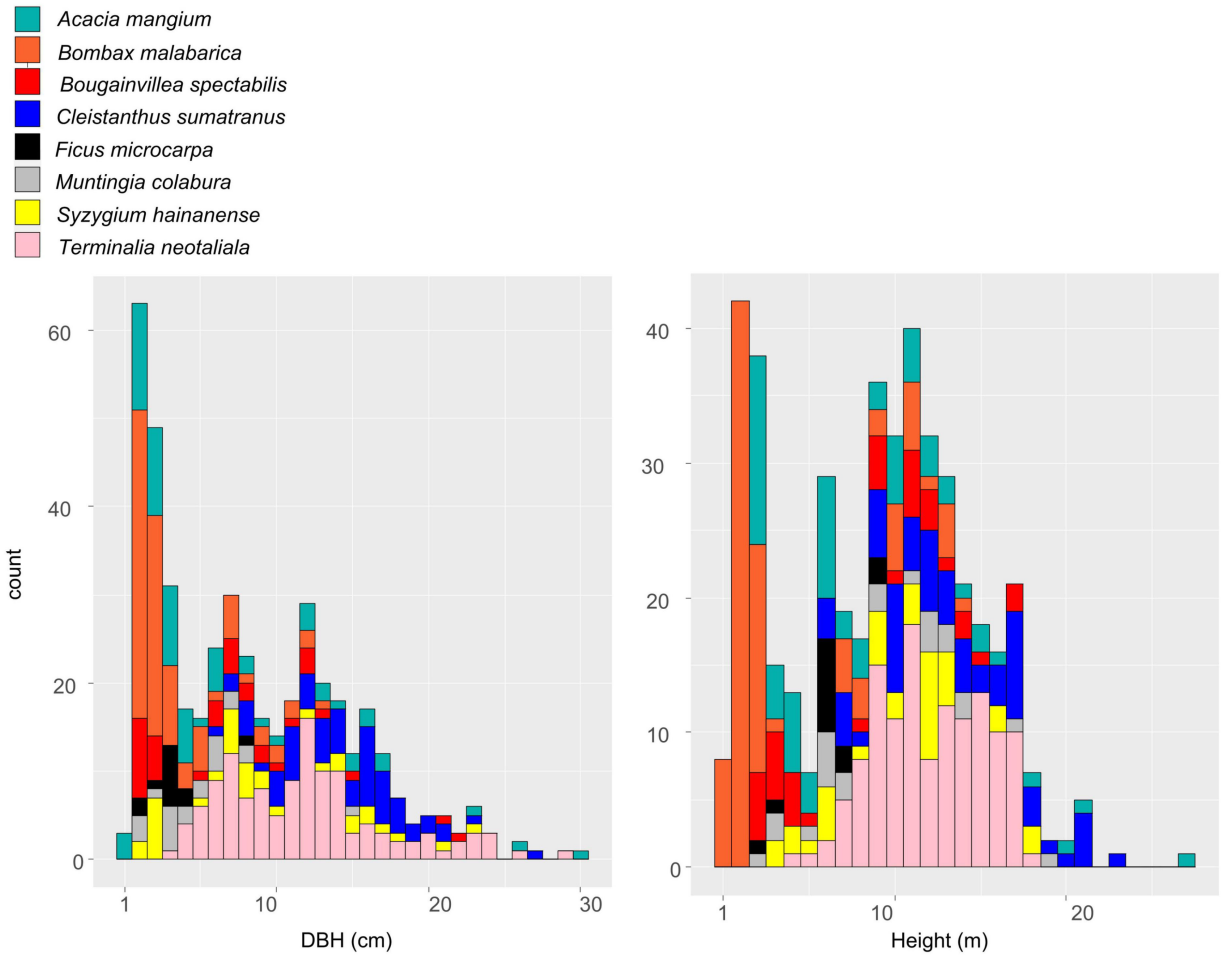
The differences in height (m) and diameter at breast height (DBH, cm) among eight selected tree species (*Acacia mangium*, *Bombax malabarica*, *Bougainvillea spectabilis*, *Cleistanthus sumatranus*, *Ficus microcarpa*, *Muntingia calabura*, *Syzygium hainanense*, and *Terminalia neotaliala*) in BPL after reforestation. Measurements were recorded in 2023.

After 7 years of the planting of the saplings of these eight tree species was performed, we found that the originally degraded landscape (comprising only barren rocks) has transformed into a forest landscape (**Figure 2**). Moreover, the survival rates of all the selected tree species were very high (90%–97%, **Figure 3**). Additionally, all the eight species could naturally recruit, which was inferred by comparing large height and DBH of the planted trees (30 m height, 30 cm DBH), compared with very small height and DBH (1 m and 1 cm, respectively) of the new trees (**Figure 4**). The large height and DBH would have been achieved by trees initially planted in 2016, whereas trees with the very small height and DBH should have been naturally recruited.

## Discussion

The standard for judging whether selected tress species are suitable for restoration in degraded ecosystem include the following aspects: 1) the original landscape has fully changed after deploying selected tree species to perform restoration (Lei et al., 2016); 2) selected tree species could adapt well to the severe environment in the degraded ecosystem, thereby having a high survival rate (Wang et al., 2020); 3) the selected tree species could recruit naturally, without requiring manual management (Holl et al., 2017). In this study, it was obvious that the originally barren land had transformed into a forest landscape after the selected tree species were deployed for restoration (**Figure 2**). Moreover, all the selected tree species had very high survival rates (**Figure 3**). In addition, all selected tree species could recruit naturally, which was indicated by presence of new trees with very small DBH and height (**Figure 4**). Furthermore, there was no need for a manual management of the reforested area since 2021. All of these results clearly indicate that the tree species selected by our protocol are suitable for reforestation in the target ecosystem.

Traditional trial-and-error-based methods have been widely used to select candidate species for ecosystem restoration, but these usually take at least one to three years to select one to five appropriate plant species (Giardina et al., 2007; Funk et al., 2008; Hall et al., 2011; Kanowski, 2014). Trait-based frameworks have been widely applied across tropical forest communities to understand variations in assemblage during a succession and at different spatial scales (Garnier et al., 2004; McGill et al., 2006; Shipley et al., 2006; Kraft et al., 2008; Zhu et al., 2013; Zhang et al., 2018). While these have established a theoretical basis for ecological restoration in degraded tropical forests, more thorough field research is needed to prove that these frameworks can be successfully applied to restore extremely degraded tropical forest ecosystems. Based on these theoretical basis, here we have provided a trait-based selection protocol that can be used to identify appropriate tree species for reforestation in degraded tropical forests. Indeed, our protocol required only 1 month to measure functional traits. Using our trait-based protocol, we successfully selected eight tree species from 60 candidates which are proven very appropriate for quickly restoring the degraded tropical mining. Our protocol is therefore effective in the selection of appropriate tree species for reforestation in degraded tropical mining.

It should be noted that a successful forest restoration must follow the ecological successional theory so that the degraded forest could recover to the originally undisturbed conditions (Funk et al., 2008). Fast-growing tree species usually dominate early forest succession (Lohbeck et al., 2014), whereas late successional slow-growing tree species gradually replace these early-successional species (Grime, 2006; Mason et al., 2012). Our current reforestation work only simulates early forest succession. Future studies are needed where we would use these eight selected tree species (*Bombax malabarica* and *Cleistanthus sumatranus*) in conjunction with our tree species selection software platform to select late successional tree species, which possess different sets of functional traits. Then, we mix-planted saplings of all selected late-successional tree species in the understory of these eight species, which may gradually make late-successional tree species to replace these eight species. This may facilitate to finally recover to originally late-successional tropical rainforest.

## Conclusions

Our native plant species selection protocol and the software program could help to identify appropriate tree species for reforestation in degraded tropical mining. The efficacy and the speed with which our methodology works are important for the timeliness of ecosystem restoration in highly degraded and vulnerable areas.

## Data availability statement

The raw data supporting the conclusions of this article will be made available by the authors, without undue reservation.

## Author contributions

CX: Conceptualization, Data curation, Formal analysis, Methodology, Validation, Writing – original draft, Writing – review & editing. HY: Conceptualization, Investigation, Methodology, Supervision, Writing – original draft, Writing – review & editing. CC: Conceptualization, Data curation, Investigation, Methodology, Validation, Writing – original draft, Writing – review & editing. HZ: Conceptualization, Data curation, Investigation, Methodology, Project administration, Software, Validation, Writing – original draft, Writing – review & editing. CW: Formal Analysis, Methodology, Validation, Writing – original draft, Writing – review & editing.
