# Supplementary material for: A quick and effective trait-based protocol for selecting appropriate native plant species for the reforestation of degraded tropical mines
Source: Front Plant Sci. 2024 Aug 14;15:1456740. doi: 10.3389/fpls.2024.1456740 (PMC11349678; doi:10.3389/fpls.2024.1456740)

**Table S1.** Functional trait measurements

*Measurement of leaf anatomical traits*

20 mature and sun-exposed leaves were collected from five independent individuals. All leaf samples were progressively dehydrated in an ethanol series (50%, 70%, 85%, 95%, and 100%) and infiltrated with warm paraffin. Then thickness of leaf, upper epidemic, lower epidemic, spongy tissue, and palisade were measured with a Leica DM2500 light microscope (Leica Microsystems Vertrieb GmbH, Wetzlar, Germany). The density of stomata was measured using a razor to slice the abaxial epidermis. All the sections were mounted on slides and observed under a Leica DM2500 microscope.

*Measurement of* maximum *photosynthesis rate, transpiration rate and stomatal conductance*

## Measurements of maximum photosynthesis rate, transpiration rate and stomatal conductance were conducted between 9:00 and 11:00 on sunny days with a Li-6400 portable photosynthesis system (Li-6400, Li-Cor, Lincoln, Nebraska, USA). Based on preliminary trials, photosynthetic photon flux density was set at 1500 μmol m^-2^ s^-1^ to ensure that light-saturated photosynthetic rates were measured for *Bombax ceiba* and *Bridelia tomentosa*. Ambient CO_2_ and air temperature were maintained at 390 μmol mol^-1^ and 28°C, respectively. Before data were recorded, leaves were exposed to the above conditions for about 5 minutes to allow photosynthetic parameters to stabilize.

## *Measurement of specific leaf are and leaf dry matter content*

Leaves were collected from the six smallest and six largest individuals of each species. Leaf chlorophyll concentration was evaluated as the average of three points on each leaf by a portable chlorophyll meter (SPAD 502, Plus Chlorophyll Meter, Konica Minolta, USA), based on a significant positive relationship with total chlorophyll. Leaf size (cm^2^) was determined using a scanner (CanoScan LiDE 700F), and analyzed with an image processing software (ImageJ, version 1.43u, National Institute of Mental Health, Bethesda, Maryland, USA). Leaf lamina thickness was measured on each side of the main vein at the widest part of each leaf (to avoid major veins) using a micrometer.

*Measurement of leaf hydraulic conductivity and leaf turgor loss point*

## Leaf-bearing branches from three to five individuals of each species were harvested and transported to the laboratory where the basal ends of the branches were immersed in distilled water and re-cut. The branch samples were rehydrated until leaf water potential was greater than -0.05 MPa. Then 9-15 fully expanded and health leaves were selected to measure leaf hydraulic conductivity using rehydration technique following Brodribb & Holbrook (2003). We also selected another 9-15 fully expanded and health leaves to obtain the initial fresh mass and then immediately placed in a pressure chamber to determine the initial water potential. Leaf mass and water potential were measured periodically during slow desiccation in the laboratory. Finally, leaves were oven-dried for 72 h at 70°C to determine their dry mass. Leaf turgor loss point was determined using a pressure-volume relationship analysis program developed by .

**References**

Brodribb, T.J., Holbrook, N.M., 2003. Stomatal Closure during Leaf Dehydration, Correlation with Other Leaf Physiological Traits. Plant Physiol. 132(4), 2166-2173. https://doi.org/10.1104/pp.103.023879.

Schulte, P., Hinckley, T., 1985. A comparison of pressure-volume curve data analysis techniques. J. Exp. Bot. 36(2), 1590-1602. https://doi.org/10.1093/jxb /36.10.1590.

**Figure S1.** Example image of the Graphic User Interface of the tree species selection software platform.


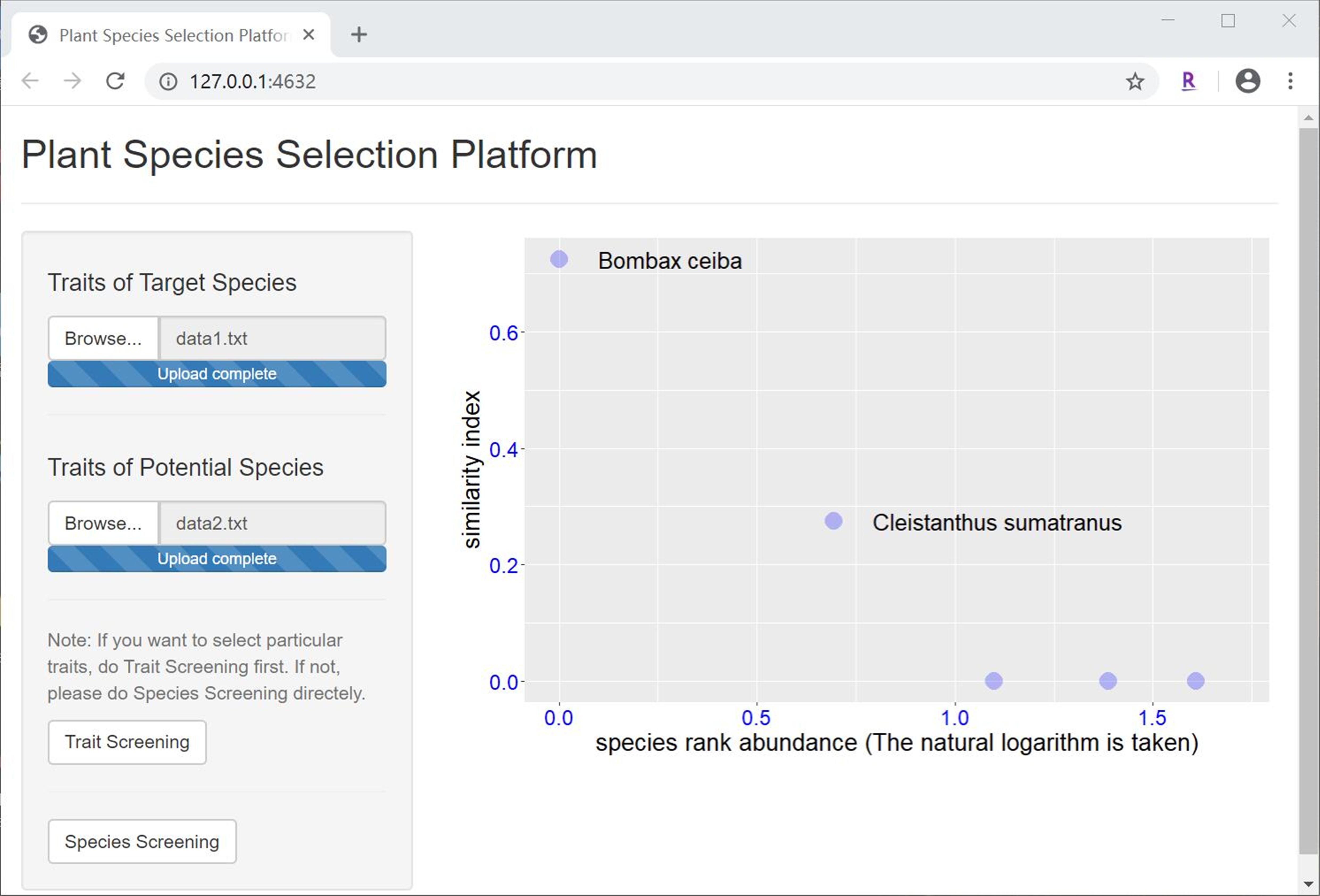

Supplement: Supplementary file 1 [file Datasheet1.doc]
